# Prepenile Scrotum- An Extreme Form of Penoscrotal Transposition

**Published:** 2013-10-01

**Authors:** Anjan Kumar Dhua

**Affiliations:** Pushpanjali Crosslay Hospital, Vaishali, Ghaziabad

**Dear Sir**

A full-term newborn male baby was brought with abnormal genitalia. The baby was a product of non-consanguinous marriage and the antenatal check ups and sonography were unremarkable. The boy had passed urine and meconium before arrival. Baby was warm, pink and active. There was no abnormal facies. Limbs and spine were normal. Local examination (Fig. 1) revealed a normal phallus with meatus at the tip of glans but it was positioned in the perineum between the scrotum and anus. The hemiscrotums were well developed and contained testis on either side. The anus was patent and its site was normal. Sonography of spine,cranium and urinary tracts showed no pathology. Echocardiogram was also normal. 

**Figure:**
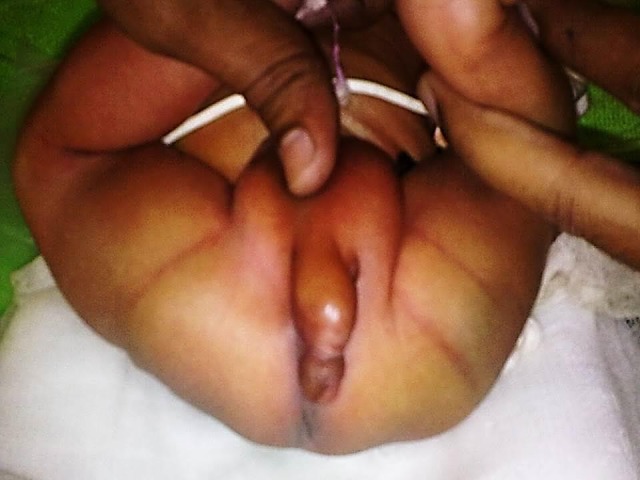
Fig. 1: Image showing a well-developed phallus in between the scrotum and anal opening.

A pre-penile scrotum or a complete penoscrotal transposition [CPST] is a rare congenital anomaly where the phallus lies between the scrotum and anus. Less than 20 cases of CPST with intact scrotum are reported in the literature [1]. Most of the cases are sporadic. Usually they are associated with other major anomalies of the cardiovascular, gastrointestinal or renal system [2].The embryological explanation of this condition remains elusive. It is postulated that an abnormal positioning of the genital tubercle in relation to the scrotal swellings during the critical fourth to fifth week of gestation could affect the migration of the scrotal swellings [3]. Another school of thought suggests that the phallic tubercle is intrinsically abnormal and affects the corpora’s development which explains the flaccid and hypoplastic penis usually found in this condition [2]. Kain et al proposed that abnormality in the gubernaculum could also result in defective migration of the labio-scrotal folds leading to these kinds of penoscrotal anomalies [4]. The variety of explanations offered clearly summarizes that the complex interactions resulting in this anomaly is yet to be deciphered. 

As expected for any rare condition a standardized treatment protocol do not exist. It has to be individualized depending on the specific anatomy. Usually an operative correction is offered at 12-18 months of age [5]. Somoza et al had successfully demonstrated a 3-staged correction, 1st stage being done at 18 months of life after topical Testosterone application [1]. Our patient has been discharged with a plan to do a staged repair at around the same age.

This case is being projected to add on to the existing literature about this rare scenario and to emphasize that a search for other anomalies must be undertaken before subjecting them to corrective surgeries.

## Footnotes

**Source of Support:** Nil

**Conflict of Interest:** None

